# A Joint Scheduling Scheme for WiFi Access TSN

**DOI:** 10.3390/s24082554

**Published:** 2024-04-16

**Authors:** Zhong Li, Jianfeng Yang, Chengcheng Guo, Jinsheng Xiao, Tao Tao, Chengwang Li

**Affiliations:** School of Electronic Information, Wuhan University, Wuhan 430072, China; 2017301200337@whu.edu.cn (Z.L.); netccg@whu.edu.cn (C.G.); xiaojs@whu.edu.cn (J.X.); tt1295@whu.edu.cn (T.T.); licw980824@126.com (C.L.)

**Keywords:** time-sensitive network, WiFi, scheduling scheme, queuing model

## Abstract

In the context of Industry 4.0, industrial production equipment needs to communicate through the industrial internet to improve the intelligence of industrial production. This requires the current communication network to have the ability of large-scale equipment access, multiple communication protocols/heterogeneous systems interoperability, and end-to-end deterministic low-latency transmission. Time-sensitive network (TSN), as a new generation of deterministic Ethernet communication technology, is the main development direction of time-critical communication technology applied in industrial environments, and Wi-Fi technology has become the main way of wireless access for users due to its advantages of high portability and mobility. Therefore, accessing WiFi in the TSN is a major development direction of the current industrial internet. In this paper, we model the scheduling problem of TSN and WiFi converged networks and propose a scheme based on a greedy strategy distributed estimation algorithm (GE) to solve the scheduling problem. Compared with the integer linear programming (ILP) algorithm and the Tabu algorithm, the algorithm implemented in this paper outperforms the other algorithms in being able to adapt to a variety of different scenarios and in scheduling optimization efficiency, especially when the amount of traffic to be deployed is large.

## 1. Introduction

As the current industrial production becomes intelligent and informatized, the demand for interconnection and interoperability between industrial equipment is increasing, and it is also necessary to continuously develop network technology to meet its needs [1]. Early applications in industrial production transmission protocols are usually specialized protocols, such as TTEthernet [2], EtherCAT, PROFINET [3,4], etc. These protocols in the link layer or the application layer make specific modifications to meet the needs of specific industrial equipment for data transmission and have limitations on how they can interconnect with each other. Therefore, the IEEE 802.1 Time-Sensitive Networking Working Group proposed a new Ethernet protocol standard in 2012, which introduces time-sensitive features on Ethernet, including precise time synchronization [5], traffic scheduling [6,7,8], frame preemption [9], flow supervision [10], frame duplication and elimination [11], and network management [12], to support the requirements of real-time and deterministic data transmission and to support devices with high real-time and deterministic requirements or multiple different protocols accessing the same LAN to form a deterministic network with low jitter and latency.

With the rise of smart factories, mobile and collaborative robots, digital twins, and visual perception of swarms of unmanned aircraft, which are scenarios that need to accomplish deterministic and low-latency communication, in industrial environments [13], the current deployment of TSNs in industrial environments faces a problem: as the layout of devices on the factory floor becomes more dynamic, the wired Ethernet communication method is difficult to satisfy the demand of mobility, and the wireless communication method is more favorable than the wired Ethernet. Therefore, introducing a wireless transmission method into the TSN is a major issue facing the current TSN working group. WiFi technology has become the mainstream wireless communication method in homes, enterprises, and public places due to its high-speed rate, low cost, and easy deployment. However, as IoT application scenarios continue to expand, traditional WiFi technology is facing more and more challenges, such as network congestion in high-density environments, bottlenecks in real-time and deterministic requirements.

In the field of industrial automation, the commonly used wireless transmission protocols are WirelessHART, ISA100.11a, WIP-PA, ZigBee, and so on. These protocols also suffer from the problem of being too specialized to interconnect and meet the needs of other kinds of applications. With the progress of IEEE 802.11 [14] research, WiFi is emerging as another major option, and WiFi networks are widely used by home users as well as in some industrial scenarios. Compared with other wireless communication technologies, WiFi has the following advantages: (1) wide transmission range, with a radius of up to several hundred meters; (2) low cost and easy access to the internet and other networks; (3) high throughput; (4) good openness and scalability. The WiFi hardware platform supports the deployment of multiple applications and the modification of MAC layer protocols and can be easily customized according to one’s own needs to achieve network security, QoS support, resource management, and other functions, which is especially important in the context of Industry 4.0 open interconnection. However, WiFi is not designed for industrial applications. Due to its CSMA/CA access mechanism, the mechanism listens to the channel before data are sent, and data transmission takes place if it is idle; otherwise, it will wait for a random period of time. Moreover, communication under wireless channels inevitably faces the problem of packet loss. This approach makes the data transmission delay uncertain and is a best-effort delivery method, which makes it difficult to meet the demand for reliable transmission in areas with high network delay transmission requirements. With the continuous expansion of industrial internet application scenarios, traditional WiFi technology faces more and more challenges, such as network congestion in high-density environments, bottlenecks in real-time and deterministic requirements, etc., which require many adjustments to WiFi to meet the industrial demands, both at the protocol level and at the device level. Therefore, combining WiFi protocol with the TSN is very valuable for research and application.

The rest of the paper is organized as follows: Section 2 presents related work. Section 3 states the problem. Section 4 discusses the modeling scheme. Section 5 describes how the GE algorithm can solve the problem. Section 6 gives the experimental results. Section 7 provides the conclusion.

## 2. Related Work

### 2.1. TSN Scheduling-Related Research

**Figure 1:**
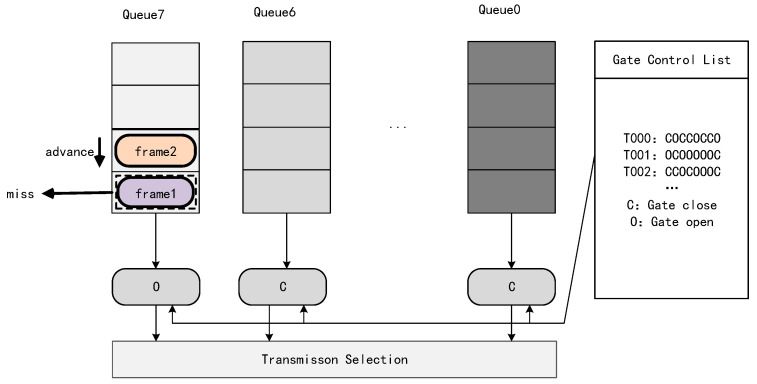
Queuing model timing disorder problem due to packet loss.

The Time-Sensitive Networks Working Group specifies the four main traffic scheduling mechanisms: credit-based shaping (CBS) as specified in IEEE 802.1Qav, cyclic queue forwarding (CQF) as specified in IEEE 802.1Qch, MAC preemption as specified in IEEE 802.1Qbu, and time-aware shaping as specified in IEEE 802.1Qbv (TAS). One of the most widely studied scheduling mechanisms is TAS. The TAS mechanism requires the computation of routing and opening and closing of gate control switches based on a given network as well as time-sensitive flows, as shown in Figure 1. When the traffic enters the switch, it enters into different queues according to the priority, and the subsequent opening and closing of the gate control switches is carried out according to the gate control switching sequence computed by the scheduling algorithm for a specified time. In ref. [15], it is stated that the solution of the gate control switch sequence is an NP-hard problem. There are more solution methods designed for this problem, in general, and they are mainly divided into two kinds: one is the ILP method which traverses the solution space to find a reasonable scheduling scheme, and the other is the ILP method.

Some representative papers are [16]. Craciunas S. S. in paper [17] develops a scheduling model for TAS and solves it using satisfiability mode theory (SMT). Ref. [17] uses the CPLEX solver to jointly solve for time slots and routing of time-sensitive flows. Another approach is the heuristic algorithm approach for solving—such algorithms do not explore the entire solution space but start generating a new solution from some initial solution and subsequently keep iterating to generate a new solution based on the new solution as well as the updated generating law, which is capable of obtaining a higher quality solution using a shorter period of time. The use of the GRASP algorithm to solve the scheduling problem of AVB streams has been proposed in ref. [18]. In [19], a method is proposed to solve the TSN scheduling problem using a genetic algorithm, which models the joint scheduling of routing time slots when all streams are of the same period. This method solves it using the GA, and the results for the transport stream spanning are improved by 31% compared with the common LS algorithm, but the solution time is too long compared with LS. In ref. [20], a greedy algorithm based on local shortest delay and a heuristic algorithm based on ant colony are proposed for solving the TSN scheduling problem, and the algorithms have better performance in terms of delay and jitter. Ref. [21] models no-wait transmission for wired TSNs with homogeneous periodic flows and optimizes the scheduling flow span using a taboo algorithm, which is shown to be effective in reducing the guard band in scheduling. An approach based on local space exploration is proposed in ref. [22], and the algorithm is able to achieve better scheduling results in scenarios with larger network sizes. Ref. [23] proposes a DoC-aware streams partitioning (DASP)-based approach, which computes the flow paths by partitioning the network, thereby reducing the conflicts among the flows, and designs an iterative integer linear programming-based scheduling (IIS) technique for incremental scheduling of flows. The method effectively reduces the conflicts of network flows and significantly improves the schedulability of flows.

### 2.2. Research Related to Wireless Time Sensitive Networks (WTSN)

In the context of the new generation of WiFi technology (IEEE 802.11be) that proposes to introduce time-sensitive transmission mechanisms in WiFi [24], WTSNs are starting to receive a lot of attention. A blueprint for WTSN development is discussed in ref. [25], which proposes that the difficulty of WTSN is that there is currently no better scheme to manage the retransmission of failed frames. A key aspect of WTSN research is the integration of WiFi. A hybrid solution combining a wired TSN with a new communication system for 802.11g WiFi is proposed in [26], which extends TSN functionality to WiFi in an interference-free environment. The solution defines a new physical layer (PHL) and a TDMA MAC layer. The drawback of this model is the low amount of traffic that can be transmitted by a WiFi node compared with the normal use implementation of WiFi. An approach to reduce the latency and jitter performance of WiFi systems in industrial automation use cases by introducing higher priority access classes at the WiFi MAC layer is proposed in [27]. The scheme enables the downlink to show good performance in transmitting TSN messages; however, non-TSN packets are not served within the required timing range. An improved Transmission of Opportunity (TXOP) scheme, S-TXOP, is proposed in [28], which reduces the latency of predictable time-sensitive traffic by providing a deterministic plan scheduling scheme for it and, also, reduces the control overhead of scheduling the uplink and downlink in IEEE 802.11ax. A solution called dynamic traffic classification is proposed in [29] to provide faster-dedicated access to the wireless medium for randomly generated packets of highly time-sensitive flows.

Overall, most of the proposed schemes for WiFi-TSNs are aimed at increasing the priority of time-sensitive flows in the WiFi MAC layer or isolating time-sensitive flows from non-time-sensitive flows. These schemes mainly discuss the traffic transmission performance after modifying the access mechanism in a single WiFi AP and do not discuss the traffic scheduling scenarios in industrial scenarios with WiFi access to the whole LAN. In this paper, based on the above work, we propose a network scheduling model based on the IEEE 802.11-based TSN and design algorithms to solve the problem by taking the industrial real-time control application traffic scheduling as the scenario that provides a new solution idea.

## 3. Problem Statement

In order to get a network that can simultaneously have the determinism and reliability of TSN and the portability of WiFi and to improve the network performance and application experience, researchers have begun to explore how to combine TSN and WiFi technologies.

**Figure 2:**
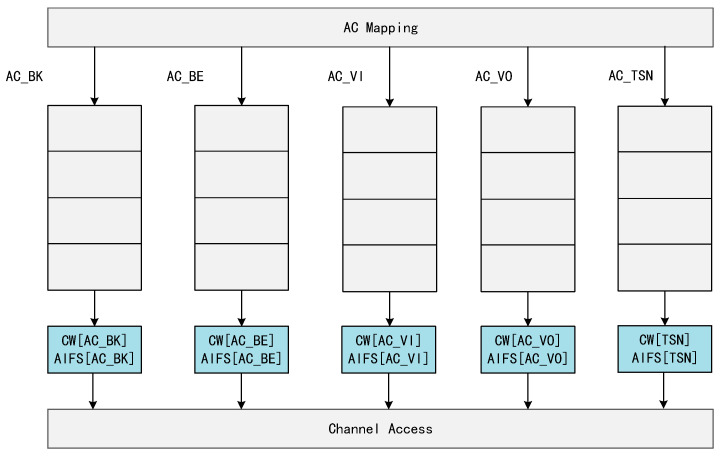
A new access category introduced in EDCA.

The main difficulties in combining WiFi and TSN are as follows:

1. The oversimplified traffic control methods in conventional Ethernet can lead to cache congestion or even blocking in the WiFi device at the receiving end, seriously affecting the transmission of time-sensitive service data streams.
2. Shared wireless links between access points (AP). Unlike Ethernet, wireless terminals experience channel contention when they are uploading data, which increases the latency and decreases the reliability of time-sensitive streams. Therefore, wireless terminals need to upload time-sensitive streams to wireless APs according to the specified time slots.
3. Queue resource division problem. In order to improve the transmission quality of delay-sensitive data, Enhanced Distributed Coordinated Access (EDCA) has been introduced in WiFi, which divides all the traffic into four different access classes, each with a different Transmission Opportunity (TXOP), arbitrated inter-frame space number (AIFSN), and minimum and maximum contention window (CWmin and CWmax), respectively. EDCA is able to increase transmission opportunities for high-priority traffic by reducing the backoff time and arbitration inter-frame space. The switches in a time-sensitive network are divided into eight priority queues, each of which is controlled by a gate control queue (GCL) that controls the queue’s transmission and wait states. This means that WiFi transmissions cannot control their QoS by queuing up data with different priorities into different queues. The sending mechanism for both is shown in Figure 1 and Figure 2.
4. Wireless link transmission uncertainty problem. Wireless links always face the problem of data transmission failure; the solution in WiFi is to introduce the ACK mechanism for retransmission to ensure the certainty of data transmission, but this method increases the delay substantially.

## 4. Description of the System Model

In this paper, we use the no-wait transmission model for traffic transmission. No-wait constraints have been widely discussed in the job shop scheduling problem, which is a well-known operations research problem, with a given set of jobs and machines, where each job consists of a set of operations that can only be executed on the machines in a given order. In addition, each machine can only process one operation at a time and explores how to obtain the minimum completion time. The no-wait constraint requires that there is no waiting time between two consecutive processes of a job, which allows each operation in a process to be processed consecutively. Under the no-wait constraint, as soon as the start time of a job is determined, its processing time on each machine as well as the completion time of the job are also determined. For control traffic with high real-time requirements, the no-wait transmission model provides the theoretically lowest latency and is the most desirable transmission scheme. In the problem of combining 802.11 with TSN, the no-wait model also has the following advantages:

(1) Time-sensitive traffic does not stay in the switch or AP, avoiding traffic congestion in the AP due to different wired-wireless transmission rates.
(2) The no-wait transmission model allows time-sensitive traffic to occupy only one queue for transmission, which avoids mutual interference between traffic flows. Since wireless transmission inevitably encounters packet loss, retransmission of time-sensitive flows can result in increased jitter and wasted bandwidth. The scenarios addressed in this paper assume that the packet loss rate of wireless transmission is within the tolerance of the control system, considering that most control applications allow for a small number of packet losses or deadline misses without significantly affecting their performance. However, in the queuing model, packet loss can cause timing disruption, as shown in Figure 1, in queue 7: if according to the transmission time scheduled by TAS scheduling, frame 1 should start transmission and frame 2 is in the waiting queue and if packet loss occurs because of the wireless transmission of frame 1, then it will lead to the early start of the transmission of frame 2, which leads to the failure of the time window scheduling and increases the jitter and delay of the transmission.

Considering that the IEEE 802.11 protocol is implemented differently from the wired TSN technology, this paper adapts the EDCA to make WiFi adaptable to the transmission of time-sensitive streams.

On the basis of no-wait scheduling, time-sensitive streams only need to occupy one queue for transmission without interfering with each other. Therefore, in this paper, we introduce a new priority AC_TSN in EDCA, as shown in Figure 2. The QoS parameters of this priority level are selected as ${CW}_{min}$ = 0 and ${CW}_{max}$ = 0, AIFSN = 0. After the WiFi access point receives the TSN packet, the AP will send out the data as soon as it senses that the channel is idle without waiting for any backoff time because the AIFSN[TSN] is zero.

Real-time networks have strict end-to-end communication delays as well as deterministic requirements, so an effective method is to use a time-triggered (TT) approach for communication, i.e., each node to be transmitted sends a message at a given time, and the switch sends the message based on the node’s streaming information by controlling the opening or closing of different queues to prevent frames from competing for the link on the network, thus effectively reducing the jitter of the stream.

The IEEE 802.1Qbv standard divides network planning into two phases: routing and scheduling. In TSNs, routing and scheduling are usually performed separately. A specific routing scheme (e.g., shortest path routing) determines the data transmission path for each TT flow in the routing phase. On the other hand, the scheduling phase determines the covariance of the switches, i.e., the timing plan for opening and closing time-controlled gates along the predetermined path. In the scheduling phase, the algorithm may not be able to find an available schedule for a path.

The resource scheduling problem for TSNs is a linear programming problem and ref. [30] proves that such problems are NP-hard. Since all the constraints elaborated in this paper are linear functions, when the scheduling objective is also linear, the problem becomes an integer linear programming (ILP) problem, which can be solved directly by an ILP solving. Although the problem can be solved directly by the ILP solver, it needs to obtain the optimal solution by traversing the entire solution space defined by the constraints, so the time complexity is high, and the algorithm performs poorly when the problem size is large. In addition, for nonlinear objective functions, the ILP is unable to solve this problem, which has greater limitations in applications.

**Figure 3:**
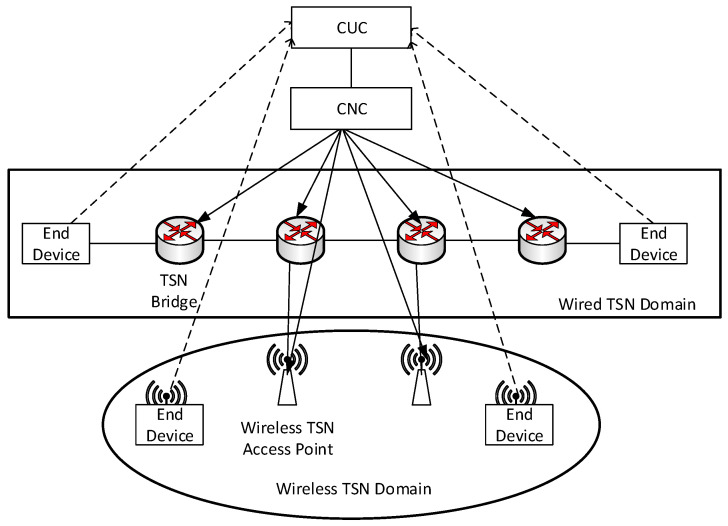
WTSN network model.

Ref. [31] gives a descriptive model of time-triggered systems, which is currently the main modeling approach for TSN scheduling models. Meanwhile, we follow the WTSN network model given in the literature [32,33], which is a network model designed based on the 802.1Qcc protocol. In this case, the centralized user configuration (CUC) unit and the centralized network configuration (CNC) unit globally configure the network as shown in Figure 3.

The CUC is responsible for collecting the requested information from the flows. The CNC communicates with the CUC and receives the communication requests from the network sent from the CUC. The CNC aggregates all the requests, calculates the route for each communication request, schedules the end-to-end transmission of each TSN flow, and finally sends the computed scheduling to each TSN bridge. The process of collecting information and scheduling flows for this network is as follows:

First, CNC collects all the node parameters of the network, including switches, AP bandwidth, and network topology.

Second, the CUC collects all traffic access requests.

Third, the CUC sends all traffic request information to the CNC, and the CNC calculates the scheduling result based on the network parameter information.

Fourth, the CNC configures the GCL and the routing table for each switch in the network and informs the AP of the sending time of each traffic flow, and the AP will inform the stations accessing the AP of the time slot allocation information by periodically broadcasting the beacon frames.

Fifth, the device in the terminal sends the traffic at the specified time.

The network model is abstracted as a graph G(V,E) where V represents the set of network nodes (TSN switches, wireless APs, terminals), and E represents the set of communication links connecting the nodes, including wireless links and wired links. The communication link between nodes $v_{m}{,v}_{n}$ is defined as

(1) $$e_{v_{m}{,v}_{n}}=<c,d,mt>v_{m}{,v}_{n}\in V$$

where c denotes the bandwidth capacity of the link and d denotes the propagation delay of the link. mt denotes the time granularity of the processing between the nodes at both ends of the link. In addition to the propagation delay of the link, the processing delay of the data by the switch or AP also needs to be taken into account. For simplicity of analysis, in this paper, it is assumed that the processing delay of all nodes, the propagation delay of links, and the time granularity of link processing are equal, denoted as $d_{proc},d_{prop},mt$, respectively.

Denote the set of all TT flows by F:

(2) $$F=\left\{f_{i},\;i\in \left[0,N-1\right]\right\}$$

where N is the number of TT flows to be scheduled, $f_{i}$ is represented by the tuple:

(3) $$f_{i}=\left(src,dest,T,route,D,IT,L\right),i\in \left[0,n-1\right]$$

where src denotes the sending node of the stream, dest denotes the destination node to which the stream is sent, T denotes the period of the stream, route denotes the path of the stream through the network, and it is stored in an array with the elements of the array as links. D denotes the maximum delay that can be tolerated by each transmission instance of the stream. IT denotes the injection time of the stream $f_{i}$ under one network scheduling cycle. L denotes the length of the time-triggered stream. Using $len(f_{i}.route)$ to denote the number of hops that stream $f_{i}$ transmits to the target node, the transmission time of the stream $f_{i}$ on its link $f_{i}.route\left[j\right],j\in [1,len\left(f_{i}.route)\right]$ can be expressed as:

(4) $$d_{f_{i}.route\left[j\right]}=\frac{f_{i}.L}{c_{f_{i}.route\left[j\right]}}$$

Thus, the delay accumulated by the stream $f_{i}$ to complete propagation over the link $f_{i}.route\left[j\right],j\in [1,len(f_{i}.route)]$ is:

(5) $$D_{f_{i}.route\left[j\right]}=j\times \left(d_{proc}+d_{prop}\right)+\sum_{k=0}^{j}d_{f_{i}.route\left[k\right]}$$

The scheduling period of the network is defined as the least common multiple of all flow periods:

(6) $$T_{sche}=LCM\left(f_{i}.T\right),i\in \left[0,n-1\right]$$

Each component in the network repeats the act of sending, forwarding, and receiving according to this cycle, so the scheduling problem simplifies how the scheduling is performed under this scheduling period. $T_{sche}$ is the minimum time interval over which the schedule repeats. If $T_{sche}$ is the length of this interval, then for any integer k>0, scheduling in $\left[0,T_{sche}\right]$ is the same as scheduling in $\left[kT_{sche},(k+1)T_{sche}\right]$. For a set of periodic tasks activated synchronously at the moment t=0, the hyperperiod is given by the least common multiple of the period, as shown in Equation (6).

During the $T_{sche}$ time, the stream $f_{i}$ sends the following number of frames:

(7) $$N_{f_{i}}=\frac{T_{sche}}{f_{i}.T}$$

The flow $f_{i}$ has been successfully deployed in the network if all $N_{i}$ frames of the flow $f_{i}$ are sent within $T_{sche}$ time and received within the specified delay.

Traffic transmission in the network will be subject to a number of conditions to ensure that the flow can complete the transmission, the following are the core constraints of the flow in the network to achieve no-wait transmission. Additional constraints can be added as needed.

1. Time slot reservation constraints

A certain time slice needs to be reserved between the WiFi and the stations for beacon frame decentralization, which is a type of management frame that is used for broadcasting control information within the BSS, completing time synchronization between APs and STAs, etc. The time slot reservation constraint ensures that beacon frames can be decentralized. Time slot information decentralization requires network-wide synchronization, so in the time slot structure designed in this paper, the first $T_{Beacon}$ time of each transmission cycle is used to perform beacon frame decentralization and, therefore, cannot be used to transmit data. The mathematical form of the constraint is: ∀$f_{i}\in F$:

(8) $${T_{Beacon}<f}_{i}.IT$$

2. Injection time constraints

Considering the periodicity of TT streams, it is ensured that the streams can send the corresponding transmission instances in each cycle. There must be a frame sent within one cycle time of the stream; therefore, it is chosen to limit the injection time of the stream $f_{i}$ to 0 to $f_{i}.T$. This limit can reduce the search space. ∀$f_{i}\in F$:

(9) $${0\leq f}_{i}.IT<f_{i}.T$$

3. Transmission instance sending time constraints

Once the injection time is determined, the sending moment of subsequent transmission instances can be derived in conjunction with the flow period. Equation (10)

(10) $$f_{i,j}^{route\left[k\right]},i\in \left[0,N-1\right],j\in \left[0,N_{i}-1\right],k\in \left[0,len\left(f_{i}.route\right)-1\right]$$

denotes the jth transmission instance of the stream $f_{i}$ on its kth transmission path where $len(f_{i}.route)$ denotes the number of links through which the stream $f_{i}$ is transmitted to the target node. $f_{i}^{route\left[k\right]}.IT$ denotes the injection time of the initial transmission instance of the stream on the link $f_{i}.route[k]$, and $f_{i,j}^{route[k]}.IT$ denotes the time of the start of transmission of the jth transmission instance of the stream on the link $e_{v_{m}{,v}_{n}}$, and the relationship between the two is shown below:

$$f_{i,j}^{route\left[k\right]}.IT=f_{i}^{route\left[k\right]}.IT+j\times f_{i}.T,$$

(11) $$\;i\in \left[0,N-1\right],j\in \left[0,N_{i}-1\right],k\in \left[0,len\left(f_{i}.route\right)-1\right]$$

4. Flow transmission constraint

The flow transmission constraint specifies a constraint on the start time of a stream transmission on a given path. Since the transmission considered in this paper is a no-wait transmission, the constraint is as follows:

$$\forall f_{i}\in F,\;k\in [0,len(f_{i}.route)-2]$$

(12) $$f_{i}^{route\left[k+1\right]}.IT=f_{i}^{route\left[k\right]}.IT+d_{f_{i}.route\left[k\right]}+d_{proc}+d_{prop}$$

5. Link conflict-free constraints

The link conflict-free constraint requires that any two streams on the same link cannot overlap with each other during transmission. Since this model takes into account the no-wait scheduling, each switch receives a frame and immediately forwards it after the processing delay $d_{proc}$. Then, any stream when its injection time is determined is able to determine the sending time of the stream on each switch. On the common link of the two streams, there are two kinds of relationships between any pair of transmission instances of the stream $f_{i}$ and the stream $f_{j}$: one is that the transmission instance of $f_{i}$ is transmitted first and then the transmission instance of $f_{j}$ is transmitted; the other is that the transmission instance of $f_{j}$ is transmitted and then the transmission instance of $f_{i}$ is transmitted. We denote by $D_{f_{i}.route\left[k\right]}$ the accumulated delay when the flow $f_{i}$ finishes the propagation on the link $f_{i}.route\left[k\right]$. Then according to the constraint in Section 4, there is:

(13) $$D_{f_{i}.route\left[k\right]}=k\times \left(d_{proc}+d_{prop}\right)+\sum_{l=0}^{k}d_{f_{i}.route\left[k\right]}$$

Thus, the link conflict-free constraint is expressed as:

$${\forall f}_{i},f_{j}\in F,f_{i}\neq f_{j},\forall m,n,s.t.f_{i}.route\left[m\right]=f_{j}.route[n],\;\forall a\in \left[0,N_{f_{i}}-1\right],\forall b\in \left[0,N_{f_{j}}-1\right]:$$

(14) $$f_{i}.IT+b\times f_{i}.T+D_{f_{i}.route\left[m-1\right]}+d_{proc}+d_{prop}\geq \;f_{j}.IT+b\times f_{j}.T+D_{f_{j}.route\left[n\right]}\;or\;f_{j}.IT+b\times f_{j}.T+D_{f_{j}.route\left[n-1\right]}+d_{proc}+d_{prop}\geq \;f_{i}.IT+a\times f_{i}.T+D_{f_{i}.route\left[m\right]}$$

6. Delay constraints

A delay constraint requires that the delay accumulated by a flow propagating along a path does not exceed its specified delay limit: ∀$f_{i}\in F$:

(15) $$f_{i}.IT+D_{f_{i}.dest}\leq f_{i}.D$$

where $D_{f_{i}.dest}$ denotes the cumulative delay of the stream $f_{i}$ transmission to the target node:

(16) $$D_{f_{i}.dest}=len\left(f_{i}.route\right)\times \left(d_{proc}+d_{prop}\right)+\sum_{k=0}^{len\left(f_{i}.route\left(j\right)\right)-1}d_{f_{i}.route\left[k\right]}$$

7. Flow Span Constraints

The flow span, which is represented by the time at which the last transmission instance of a stream completes its transmission, is denoted by the flow span of the stream $f_{i}$:

(17) $$C_{f_{i}}=f_{i}.IT+D_{f_{i}.dest}+{(N}_{f_{i}}-1)\times f_{i}.T$$

The maximum value of the flow span is expressed as

(18) $$C_{max}=max\{C_{f_{i}}|f_{i}\in F\}$$

If the network is still delivering the traffic from the previous cycle before the start of a cycle of scheduling, then there is a possibility that the traffic may be preempted by beacon frames or cause the sending of data frames to be queued up in the network. In order to avoid this problem, the flow span should be restricted to be within the network scheduling cycle [34]; thus, the flow span constraint is formulated as:

(19) $$C_{max}\leq T_{sche}$$

The scheduling target should be selected with reference to the requirements of the actual scenario application, such as a business requiring the sampling period to be as stable as possible, the total transmission delay to be as small as possible, and so on. The scheduling function can be adjusted according to the characteristics of the business focus. In this paper, we choose to minimize the flow span as the goal of flow scheduling, which is

(20) $$minimize\;C_{max}$$

There are several reasons for choosing flow span as the optimization objective. First, the scheduling adopted in this paper is no-wait scheduling, which is a theoretical scheduling scheme with the lowest latency, so there is no need to select latency as the optimization objective. Second, it is mentioned in ref. [21] that switching the gate control switches several times can lead to an increase in the number of protection bands of the flow and, thus, reduce the transmission efficiency, so by optimizing the flow span, the flow can be made as compact as possible, which reduces the frequency of gate control switch switching and increases the transmission efficiency. And since the flow span is optimized, the links within the network have more room to transmit resources continuously, which makes the network more capable of transmitting best-effort traffic. Third, wireless accesses will have more device changes compared with wired accesses, and lower flow spans will allow newly added traffic to be scheduled faster according to the greedy algorithm.

## 5. Algorithm Design

In this paper, we propose a scheme based on the greedy strategy distributed estimation algorithm (GE) to solve the scheduling problem. Estimation of distribution algorithms (EDA) is a statistically based optimization method that continuously updates the probabilistic model by building a probabilistic model from a population macroscopic point of view for describing the distribution information of the solution in the search space, learning the parameters of the sample distribution, generating new samples, and evaluating their fitness to find the optimal solution. Compared with genetic algorithms, EDA has stronger global search capability and faster convergence speed. The implementation process of the standard EDA algorithm is as follows [35]:

1. Initialize a population of candidate solutions ${\{x}_{i}\},i\in [1,N]$.
2. Repeat Step 3–Step 5 and loop until the termination criterion is reached.
3. According to the size of fitness, select M individuals from ${\{x}_{i}\}$, where (M<N)
4. Based on the statistics of the M individuals selected above.
5. Generate a new population ${\{x}_{i}\},i\in [1,N]$ from the statistics.

EDA has been shown to achieve good results in job shop scheduling problems [36]. In this paper, we take the approach of a distribution estimation algorithm to solve the job shop scheduling problem to design a scheduling algorithm to realize the no-wait wireless TSN scheduling algorithm. In this algorithm, we first give the information about the network topology and the streams that need to be scheduled and generate the scheduling order through EDA. Based on this order, we find the minimum stream injection time for each stream according to the greedy strategy until all the streams have been scheduled. This method significantly reduces the search space of the problem, and the algorithm is able to approximate the minimum flow span according to the proofs in ref. [37]. The algorithm is shown in Algorithm 1.
**Algorithm 1:** Inject Time Based on Flow Sequence.Input: Flow information F,
   Network topology model G(V,E),
   Sequence of flow Seq(F) generated by EDA
Output: Flow span $C_{max}$1: $C_{max}$ ← 0
2: for each flow in Seq(F):
3:  $startTime=Earliest\;possible\;start\;time(f_{i}.IT)$
4:  $C_{max}$ ← $max(C_{max},f_{i}.IT+D_{f_{i}.dest})$
5: endfor
6: return $C_{max}$


The function ‘Earliest possible start time()’ finds the minimum flow start time by generating a constraint expression based on the flows already deployed in the current network and finding the minimum flow start time based on the link’s time granularity mt as the smallest unit.

The algorithm for applying EDA to generate the flow order is shown below:

1. Stream order-based codecs

The method proposed in this paper uses scheduling stream order as the goal of EDA computation. It is assumed that there are a total of $\left|F\right|$ streams to be scheduled, and the number of all possible species of stream ordering reaches $\left|F\right|!$ number of streams. The size of the solution space rises sharply after the size of the streams reaches a certain level, based on which we use EDA to search the solution space. In this paper, we use a vector:

(21) $$X=\left(x_{1},x_{2},\ldots ,x_{\left|F\right|}\right)$$

to represent the stream scheduling order, the number of elements of this vector is |F|. Its internal elements are numbers from 1 to $\left|F\right|-1$ that do not repeat each other, and any element $x_{i}$ in the vector represents the *i*th position in the scheduling queue of the stream $f_{x_{i}}$.

2. Initialize the probability matrix

Unlike genetic algorithms with crossover and mutation, EDA uses a probability matrix for sampling to generate offspring, and in this paper, the *k*th generation probability matrix is expressed in the following form:

(22) $$P\left(k\right)=\left\{p_{ij}\right\}i,j\in \left[1,\left|F\right|\right]$$

where $p_{ij}$ denotes the probability that stream $f_{i}$ is placed at the position j, i.e., P(k) is set as a metric used to indicate how the streams are better ordered under the current and iterative information.

The initial probability matrix is set as:

(23) $$P\left(0\right)=\left\{p_{ij}=\frac{1}{\left|F\right|},\forall i,j\in \left[1,\left|F\right|\right]\right\}$$

This matrix indicates that the ordering of the streams in the initial state is randomly generated without preference.

3. Generate children through the probability matrix

In order to reasonably generate individuals on the basis of the probability matrix, this paper adopts the roulette way to sample the matrix to generate individuals. The specific method is to traverse each column of the probability matrix, add up the probability of the column, select the flow placed in the position through the roulette way, and set all the rows to zero. Using this method, a flow scheduling order can be obtained, which is continuously followed to generate a population containing *N* individuals:

(24) $$Pop\left(k\right)=\left\{X_{1}\left(k\right),X_{2}\left(k\right),\ldots ,X_{N}\left(k\right)\right\}$$

4. Calculate the fitness of children

With the flow-ordered population obtained in Step 2, the fitness is calculated for each individual in the population according to Algorithm 1, and *M* individuals are selected to form a set according to the size of the fitness:

(25) $$S\left(k\right)=\left\{X_{u_{1}}\left(k\right),X_{u_{2}}\left(k\right),\ldots ,X_{u_{M}}\left(k\right)\right\}$$

Subsequently, the best-fit individuals are updated based on this set.

5. Probability matrix update method

Update the statistics of these M individuals selected above according to Heb’s rule:

(26) $$P\left(k+1\right)=\left(1-\alpha \right)P\left(k\right)+\alpha \frac{1}{M}\sum_{l=1}^{M}I^{u_{l}}\left(k\right)$$

where 0<α<1 is the learning rate. $I^{u_{l}}(k)$ is an indicator matrix calculated from the individual $X_{u_{l}}\left(k\right)$, representing the probability that flow $f_{i}$ occurs before or at the position j of the sequence vector of the kth generation flow scheduling sequence. It is computed as follows:

(27) $$I_{ij}^{u_{l}}\left(k\right)=\left\{\begin{array}{c} 1\;\;\;if\;f_{i}\;appears\;before\;or\;in\;position\;j\\ 0\;\;\;else\; \end{array}\right.$$

6. Loop

Jump to Step 3 and repeat the loop until the loop termination condition is reached.

The entire algorithm flow is shown in Algorithm 2.
**Algorithm 2:** Flow Sequence Generator Based on EDA.Input: Flow information F,
   Network topology model G(V,E)
Output: minimum flowspan best1: P= Initial probability matrix(F)
2: best = max fitness(population)
3: while Iterations < maxIterations do:
4:  selection result = selection(population)
5:  P = update probability model(selection result)
6:  If max fitness(selection result) > best:
7:    best = max fitness(selection result)
8:     population = sampling(P)
9:  Iterations = Iterations + 1
10: EndWhile
11: return best

## 6. Evaluation of Experimental Results

### 6.1. Description of Experimental Conditions

To evaluate the algorithms designed in this paper, we built a Python-based (Python 3.10.5) simulation platform and used NetworkX 2.8 [38] developed in Python as a network-building and generating tool. To make comparisons, we also designed two different comparison algorithms based on the scenarios in this paper, which are the Tabu-based algorithm designed in reference [21] and the ILP-based algorithm in reference [16] for the ILP-based approach. All experiments were run under an Intel Core™ i7-6850K CPU processor with 3.60 GHz and 64 GB RAM.

**Figure 4:**
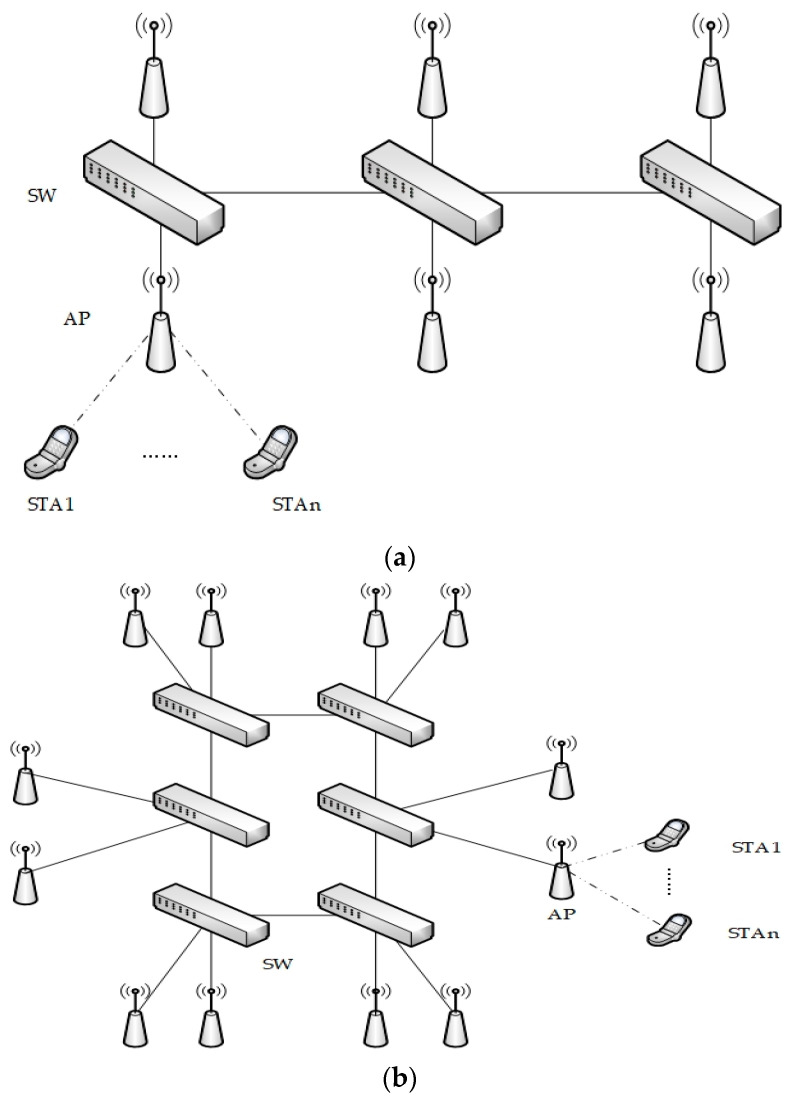

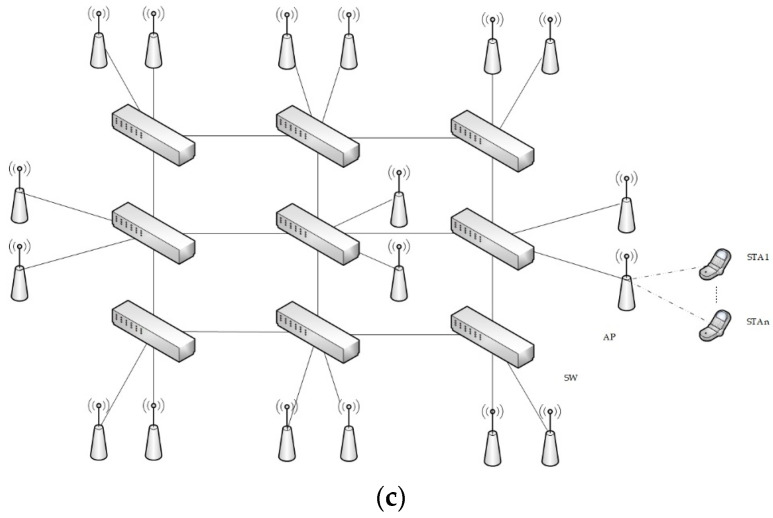
Network topology diagram schematic: (**a**) linear; (**b**) ring; (**c**) grid.

We designed three different network topologies: linear, ring, and grid. The switch connections of all three network topologies are linear in structure; each switch connects to two WiFi nodes, and several stations are connected under the WiFi nodes, as shown in Figure 4. The linear topology has 3 switch nodes and 6 WiFi nodes; the ring topology has 6 switch nodes and 12 WiFi nodes; and the grid topology has 9 switch nodes and 18 WiFi nodes. After selecting the topology, we start generating the flow request. First, we select a period from [10 ms, 20 ms, 40 ms] as the flow. Subsequently, we select the corresponding number from [0.5 ms, 1 ms, 2 ms] as the latency requirement for its flow request and randomly select two different endpoints in the network as the flow sending site and receiving site. Next, these conditions are calculated to generate the constraints and imported into the algorithm for computation. The experimental conditions are shown in Table 1.

### 6.2. Performance Evaluation of the Algorithm in This Paper

**Figure 5:**
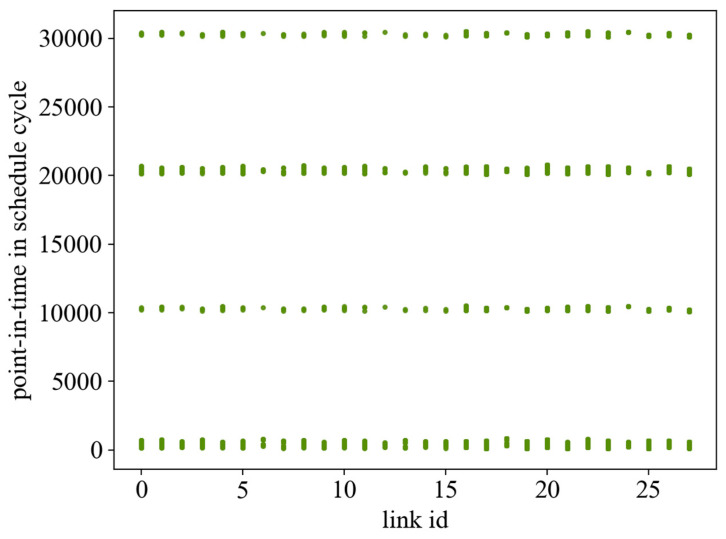
The occupancy effect of the GE algorithm scheduling result on the link.

**Figure 6:**
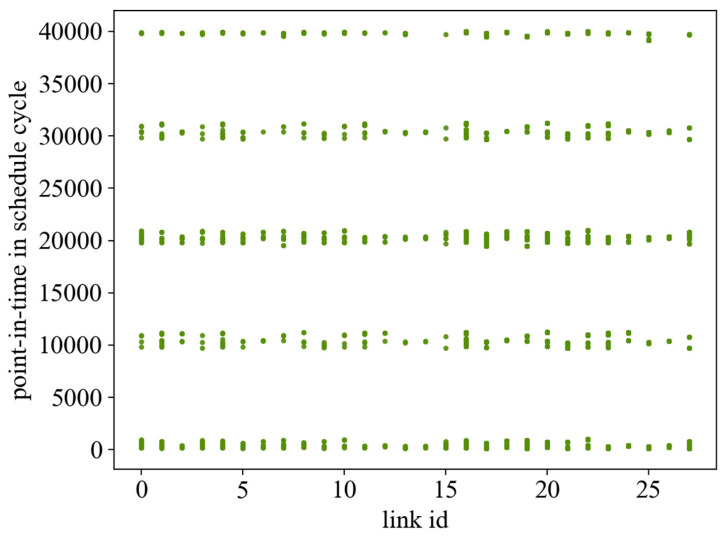
The occupancy effect of the ILP algorithm scheduling result on the link.

The searching ability of EDA is greatly affected by the learning rate α. After many experiments, the learning rate is taken as 0.2. Under this condition, this paper first gives the scheduling results given by the EDA and ILP algorithms in the case of 60 streams and the linear network topology, as shown in Figure 5 and Figure 6, where the horizontal axis denotes the links—where links 0–15 are the wired links and links 16–27 are the wireless links. The vertical axis represents the occupancy of link resources under the scheduling cycle. Comparing the two results, it can be seen that the ILP algorithm does not take the flow span as the optimization objective but only searches for the existence of the solution under the given constraints, and the scheduling result is looser in the whole network, which results in frequent door control switches and wastes the bandwidth. With a flow span of 40,000 μs., the algorithm in this paper has a more compact scheduling result, which effectively reduces the openings and closings of the door control switches and has a flow span of 30,344 μs.

**Figure 7:**
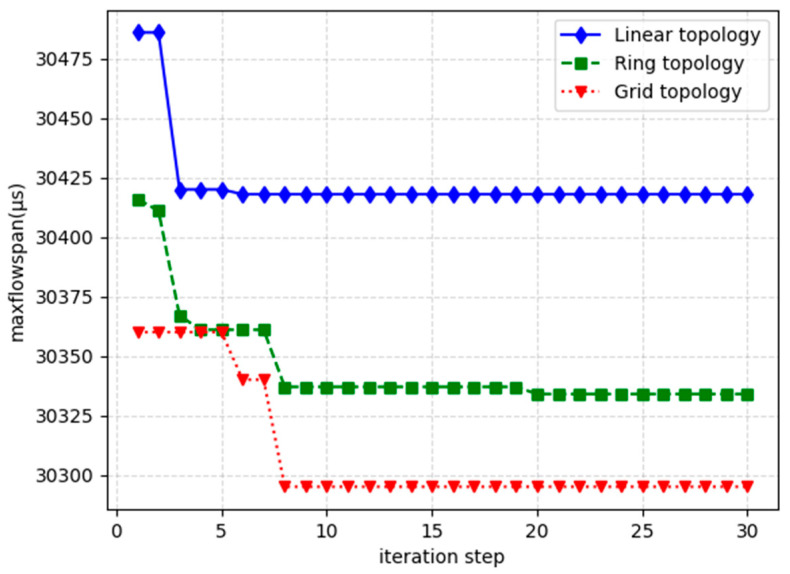
Schematic diagram of the variation of the flow span with the number of iteration steps for different network topologies.

**Figure 8:**
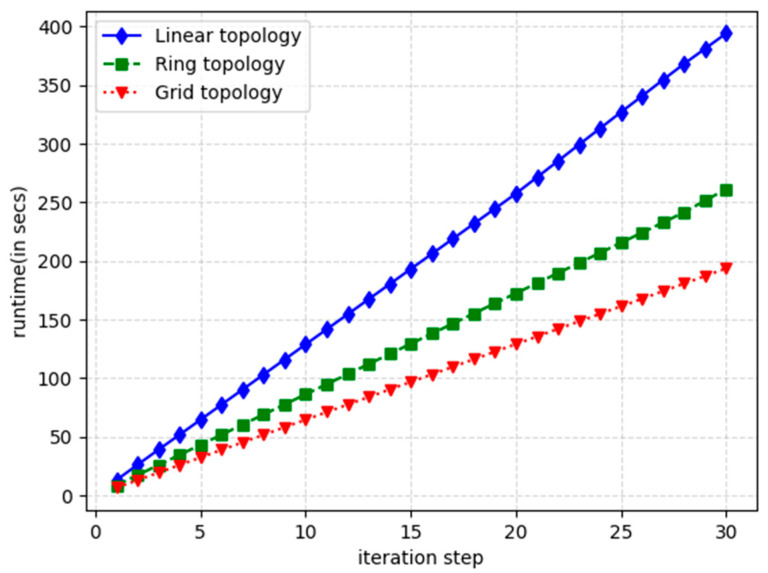
The trend of solution time with the number of iteration steps for different topologies.

To evaluate this paper’s algorithm in detail, this paper generates 60 streams under 3 different network topologies and observes the process of the GE algorithm in solving the stream span under different iteration steps as well as the time it takes. Figure 7 gives the optimization effect of the flow span with iteration steps under different topologies and Figure 8 provides the trend of solution time with iteration steps under different topologies. From the figure, it can be seen that for the same number of streams, the linear network topology solving result has the largest stream span, followed by the toroidal network topology, and the mesh network has the smallest span. In terms of network solving time, the linear network topology has the longest solving time, followed by the ring network topology and the mesh network has the shortest time. This is mainly due to the fact that the more complex the network, the more the traffic will be dispersed in the various edge nodes of the network and the more links will be circulating in the network, so the probability of collision between the traffic is smaller, reducing the flow span and the difficulty of solving.

### 6.3. Comparison with Other Algorithms

This section compares the GE algorithm with the ILP and the Tabu algorithm. We first generate a network using NetworkX, then generate flows with different numbers of flows (from 40 to 160) on the generated network, and record the values of the three algorithms for solving the flow span under different network topologies, as well as the variation of the solving time with the number of flows. To prevent the solution time from being too long, the time is limited to 0.5 h in this paper, and if the timeout is exceeded, it is considered unsuccessful and is not labeled in the figure. In order to balance the solution time of the GE algorithm and the solution results, the number of iterations is limited to 10 in this paper.

**Figure 9:**
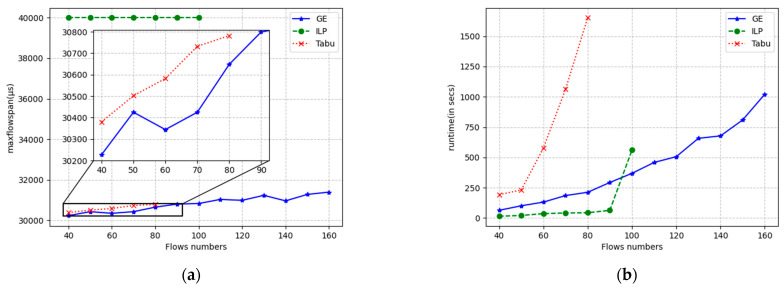
Running results of three algorithms under the linear topology with different numbers of streams. (**a**) max flow span optimization; (**b**) running time.

**Figure 10:**
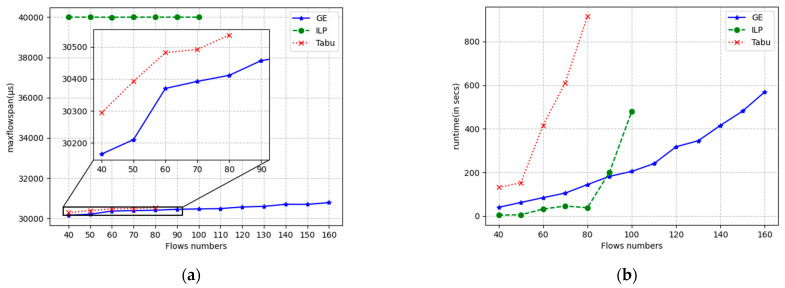
Running results of three algorithms under the ring topology with different numbers of streams. (**a**) max flow span optimization; (**b**) running time.

**Figure 11:**
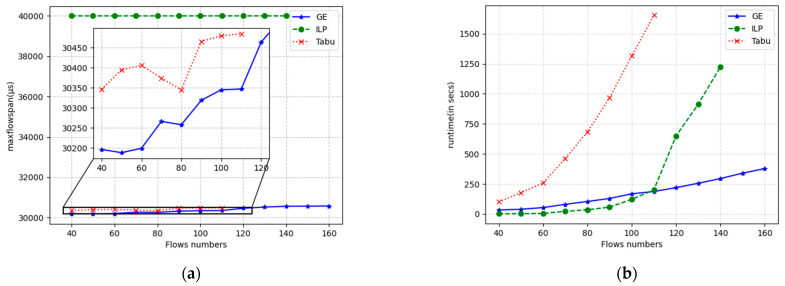
Running results of three algorithms under the grid topology with different numbers of streams. (**a**) max flow span optimization; (**b**) running time.

The results for the flow span and runtime are displayed in Figure 9, Figure 10 and Figure 11 for linear, ring, and mesh network topologies, respectively. In the linear topology, GE outperforms the Tabu algorithm by an average of 182 μs in terms of the flow span that has been solved, whereas the ILP algorithm is stable at around 40,000 μs in terms of the flow span that has been solved since it does not use the flow span as an optimization objective. When the number of flows is small, the ILP has the shortest solution time, and the GE algorithm is similar to the Tabu algorithm. As the number of flows increases, the solution time of the GE algorithm grows smoothly, while the Tabu algorithm and the ILP algorithm grow exponentially. The solution timeout is exceeded after more than 80 and 100 flows, respectively, while the GE is still able to solve the problem normally and in almost twice the number of flows compared with the Tabu algorithm with a solution time of 1000 s. The ILP algorithm can be used in the same way as the Tabu algorithm. In 1000 s, GE is able to solve almost twice the number of flows solved by the Tabu algorithm. In the ring topology, GE outperforms the Tabu algorithm by an average of 130 μs over the span of solved flows, and Tabu and ILP time out after more than 80 and 100 flows, respectively. In the mesh topology, GE outperforms the Tabu algorithm by 147 μs on average in terms of the span of flows that have been solved, and Tabu and ILP solve for timeouts after more than 110 and 140 flows, respectively. In other respects, the ring topology and the grid topology are essentially the same as the linear topology.

Combining the performance of these three algorithms under different topologies, compared with the Tabu algorithm, the GE algorithm implemented in this paper has a smoother variation of the solution time with the number of streams in each network, a shorter solution time, which yields the best solution results, and a higher efficiency in searching for the optimal solution, which is more adapted to the optimal solution of this problem. This is because the Tabu algorithm utilizes the method of searching the neighborhood, which is less efficient for this problem, and it is difficult to search for a better solution, while the EDA takes the probability-based search method, which has a higher search efficiency. Compared with the traditional ILP algorithm, the GE algorithm obtains a better solution in all the cases listed, and its solution time is closer to the ILP algorithm when the number of streams is small and significantly smaller than the ILP algorithm when the number of streams is large. This is due to the fact that the search space of ILP increases dramatically with the number of streams. Therefore, the GE algorithm is more practical than the other two algorithms.

## 7. Conclusions

In this paper, we have proposed a model as well as an algorithm for the problem of combining WiFi and the TSN. We first analyze the problems faced by the combination of WiFi and time-sensitive networks and then establish a no-wait scheduling model for time-sensitive streams based on the TAS mechanism of TSNs to address the characteristics of WiFi and time-sensitive networks, which can isolate the time-sensitive streams into a queue channel by uniformly calculating the sending time of the streams to avoid the problem of queue resource constraints when transforming WiFi and time-sensitive networks. This model can effectively avoid the problem of queue resource constraints when WiFi is converted to time-sensitive networks. On the basis of this model, this paper draws on the method of optimizing completion time in the job scheduling problem to design a distribution estimation algorithm based on the greedy strategy to optimize the network flow span so that the network can avoid dispersed, traffic scheduling, resulting in too many traffic protection bands wasting the bandwidth of the BE traffic. After experimental testing, compared with the ILP algorithm and the Tabu algorithm, the algorithm in this paper shows a higher solving efficiency and is more practical in solving the problem.

As of now, WiFi access to time-sensitive networks has not resulted in a complete solution. This paper discusses a scheme for WiFi access to time-sensitive networks in industrial internet scenarios, which has some practical value. The shortcoming of this paper is that it does not consider the link uncertainty of WiFi transmission and the handling of bursty flows in WiFi nodes. In future work, we will model the bursty flows and retransmission to find new solution schemes.

## Figures and Tables

**Table 1:** Experimental conditions.

Parameter	Value or Range
Topology	Linear, Ring, Grid
Request time period	[10 ms, 20 ms, 40 ms]
Latency requirement	[0.5 ms, 1 ms, 2 ms]
Frame size	[125 B, 250 B, 500 B]
Wireless Tx speed	100 Mbps
Wired Tx speed	1000 Mbps
Node processing time	10 μs
Link propagation delay	10 μs
Reserve transmission time for beacon frames	100 μs

## Data Availability

The data presented in this study are available on request from the corresponding author. The data are publicly available due to privacy.
